# Regulation of sleep disorders in patients with traumatic brain injury by intestinal flora based on the background of brain-gut axis

**DOI:** 10.3389/fnins.2022.934822

**Published:** 2022-10-11

**Authors:** Niu Zhanfeng, Wu Liang, Kang Jing, Bai Jinbo, Chen Yanjun, Xia Hechun

**Affiliations:** ^1^Department of Neurosurgery, General Hospital of Ningxia Medical University, Yinchuan, China; ^2^ENT & HN Surgery Department, General Hospital of Ningxia Medical University, Yinchuan, China

**Keywords:** traumatic brain injury, sleep disorder, gut microorganisms, brain-gut axis, circadian rhythm

## Abstract

**Objective:**

This study investigates whether people with sleep disorders following traumatic brain injury exhibit altered intestinal flora. The changes may allow us to gain a better understanding of the role of intestinal flora in patients with sleep disorders after traumatic brain injury, which may give us insights into curing the sleep disorder after traumatic brain injury (TBI).

**Method:**

We analyzed the intestinal microbial colony structure in the feces of the 28 patients in the normal sleep group and the sleep disorder group by 16SrDNAsequencing technology. The bioinformatics method was used to analyze the intestinal flora change in the v3-v4 region of patients with biorhythm disorder and to observe the difference between the two groups.

**Results:**

Group grouping comparison and analysis of the evolutionary cladistic map showed the intestinal flora of patients with normal sleep after TBI was mainly Bacilli and Lactobacillales, while that of patients with sleep disorders was mainly Lachnospiraceae and Bacteroidales. The histogram of group value distribution by grouping comparison and analysis showed that Lachnospiraceae, Bacteroidales, Bacteroidia, and Bacteroidetes were dominant in the sleep disorder group. A relative abundance map of species with significant differences by group grouping comparison showed the main manifestations of intestinal flora are Firmicutes, Bacilli, Lactobacillales, Streptococcaceae, and Bacteroidetes. The normal sleep group was dominated by Bacilli, Lactobacillales, Streptococcus, and Veillonella, while in the sleep disorder group, Lachnospiraceae, Bacteroidales, Bacteroidia, and Bacteroidetes were the main species. It was found that there were also significant differences in intestinal flora abundance between the two groups after TBI. After statistics processing, it was compared with the normal sleep group, Lactobacillus, Streptococcus, Oribacterium and Rothia, Actinomyces, Streptophyta, TM7-3 bacteria, and Serratia, showing a significant reduction in the sleep disorder group (*P* < 0.05). However, Odoribacter, Lachnospiraceae, and Bilophila increased significantly (*P* < 0.05).

**Conclusion:**

The sleep disorders of patients after TBI can be closely related to intestinal flora disturbance, and its internal mechanism needs further study. Intestinal flora has the potential to be a new therapeutic target.

## Introduction

Posttraumatic sleep disorder is a common problem that perplexes patients, family members, and neurosurgeons in clinical work. Only mild traumatic brain injury (TBI) has 40–65% of complaints about sleep problems (Leng et al., 2021). Early chronic sleep disorders can cause or aggravate other symptoms, such as headaches, emotional grief, depression, and cognitive impairment, and even affect the process of rehabilitation. Long-term sleep disorders can cause changes in many physical conditions, such as cardiovascular diseases and metabolic diseases, including obesity and diabetes (Ouellet et al., 2006; Jiang et al., 2019; Folmer et al., 2020).

After decades of development, many researchers have gradually deepened their research on intestinal flora and sleep disorders. Usually, we think that the abnormal transfer of intestinal flora is called flora imbalance, which has been found to exist for a long time in the body. Flora imbalance is closely related to diabetes, metabolic syndrome, fat storage, etc. Through the fecal transplantation experiment, some scholars can turn an obese mouse into a thin mouse, which is also a kind of “flora imbalance.” A large number of clinical studies have found that intestinal flora imbalance can also affect the cognitive function of patients through the “brain-gut axis” (Torres-Fuentes et al., 2017). Previous research shows a close relationship between sleep disorders and central biorhythm disorders in patients with TBI. A large number of studies have shown that the disorder of central biorhythm *in vivo* can cause disorders related to diet, exercise, stress, and so on (Mukherji et al., 2013; Gamez-Belmonte et al., 2020). The change in eating rhythm and the destruction of central biorhythm will cause time-specific changes in intestinal bacteria (Liang et al., 2014; Thaiss et al., 2014; Parris et al., 2019; Matenchuk et al., 2020). Similarly, in a large number of clinical practices, we found that patients with TBI are prone to intestinal dysfunction in addition to sleep disorders, such as diarrhea or constipation, combined with the concept of the “brain-gut axis.”

This study aims to observe whether there is a difference in intestinal flora between patients with sleep disorders after craniocerebral injury and patients with normal sleep after TBI and whether there is intestinal flora imbalance. In order to further explore the causes of sleep disorders in patients with TBI and find a treatment to solve the sleep disorders in patients with TBI.

## Materials and methods

### Patient enrollment requirements

Twenty-eight patients with TBI were selected and divided into two groups. The experimental group consisted of 14 patients with sleep disorder symptoms more than one month after TBI, called the sleep disorder group after TBI. The control group comprised 14 patients without obvious sleep disorders more than one month after TBI. All patients were quickly transferred to the General Hospital of Ningxia Medical University after injury. There was no significant difference in hospitalization time between the two groups, and none received surgical treatment. All patients’ craniocerebral CT was treated according to Marshal grade. The recruitment and sample collection processes were as follows.

This study is part of a clinical trial, “The Clinical Research on the Relationship Between Circadian Rhythm and Gut Microbiota in TBI Patients” (NCT02849028), which was approved by the General Hospital Ningxia Medical University Research Ethics Committee. All patients signed the informed consent papers.

We analyzed the characteristics of the two groups of patients (the sleep disorder TBI group and the normal sleep TBI group) from January 1, 2017, to October 1, 2017. Each group consisted of 14 patients (11 men and three women). All the patients were injured in automobile accidents. Every author could access information that could identify individual participants during or after data collection. To ensure the validity of the experimental data, the age and weight of each patient in the sleep disorder group were similar to those in the normal sleep group. Males represented 78.6% of the participants, and the average patient’s age was 41.86 ± 14.28 years (age span: 18–69 years) in the sleep disorder group and 41.64 ± 12.31 years (age span: 20–57 years) in the normal sleep group. No patients were given medication (Supplementary Table 1; Zhanfeng et al., 2019).

### Volunteer recruitment

The participants were patients diagnosed with TBI and suffering from sleep disorders or experiencing normal sleep in the neurosurgery clinic of the General Hospital of Ningxia Medical University, Yinchuan, China. The study was advertised in the department, and all patients volunteered and gave written informed consent. The patients had no history of neurological or psychiatric disorders or sleep/fatigue disturbances before the injury. They had no systemic diseases, such as heart, liver, or kidney.

### Sleep disorder diagnosis

We made the diagnosis by gathering the following information from TBI patients: sleeping time, wake-up time, the time to fall asleep, the number of awakenings during sleep, and the sleep time each night. We also needed to know about sleep-related issues, such as personal assessment of sleep quality, whether or not drugs mediated, daytime alertness and mood, and any sleep disorders caused by respiratory diseases, heart diseases, dreams and nightmares, and other discomforts. Patients had to complete the Pittsburgh Sleep Quality Index (PSQI), which includes 19 sleep quality-related questionnaires and sleep disorders for over 18 months.

The prevalence of sleep disorders and related polysomnographic (PSG) findings have been found in TBI. An electroencephalogram (EEG) is used to amplify and record the spontaneous biologic potential of the brain from the scalp with electronic instruments. It is the spontaneous and rhythmic electrical activity of the brain cell group. It is useful in monitoring sleep quality, duration, number of awakenings, and the duration of deep sleep, as well as providing a map of electrical activity in the cortex during sleep 19. Its clinical symptoms mainly include insomnia, difficulty falling asleep, recurring dreams, easy awakening, prolonged sleep, short sleep, and frequent awakenings during sleep. A neuroelectrophysiologist analyzed the EEG (Supplementary Tables 1, 2; Zhanfeng et al., 2019).

### Fecal specimen collection

The patients and their families completed the collection of fecal samples required for the experiment. The recruited patients retained fecal samples, and the fresh fecal samples were collected with 5 mL cryopreservation tubes. The scraping tools were aseptic cotton swabs to avoid fecal contamination. The mass was about 2 g, or the size was not less than 1.0 cm in diameter. The requirements for sample collection were as follows: (1) DNA concentration ≥ 20 ng/μL and 2.2 ≥ OD ≥ 1.8, DNA volume ≥ 30 μL. The sample quality meets the requirements of database construction: (2) 20 ng/μL > DNA concentration > 5 ng/μL and 2.2 ≥ OD ≥ 1.7, DNA volume ≥ 30 μL; DNA concentration ≥ 20 ng/μL and 2 ≥ OD ≥ 1.7, DNA volume < 30 μL, (3) 2 ng/μL ≤ DNA concentration ≤ 5 ng/μL and 2.2 ≥ OD ≥ 1.7, DNA volume ≥ 30 μL; DNA concentration ≥ 5 ng/μL and 1.7 ≥ OD ≥ 1.5, DNA volume ≥ 30 μL; 20 ng/μL ≥ DNA ≥ 5 ng/μL, and 2.2 ≥ OD ≥ 1.7 DNA volume < 30 μL. As far as possible, all specimens that met the requirements of specimen collection (1) and (2) samples that met the requirements were collected and sent to an 80°C refrigerator for preservation.

### Sequencing and bioinformatics analysis

First of all, after pretreatment of fecal samples frozen in a deep cryogenic refrigerator at 80°C, strict sample quality testing procedures were carried out. All samples collected in accordance with the procedure had to meet the requirements of specimen collection (1) and (2). After meeting the requirements of detection, fecal samples were further extracted by DNA, amplified by PCR, purified, mixed, and established, and the corresponding procedures were made. The stool samples of patients with sleep disorders after TBI and normal sleep after TBI were detected according to the above strict standards. Then the samples that met the requirements were sequenced by a high-throughput sequencing platform (Illumina Miseq/Hiseq). The intestinal flora in the stool samples of the two groups was sequenced, and the collected data (raw data) were obtained by bioinformatics analysis. The intestinal flora of patients with craniocerebral injury was analyzed and compared between the sleep disorder and normal sleep groups.

According to the preliminary results of intestinal flora analysis in the sleep disorder group and the normal sleep group after TBI, the following specific analysis operations were carried out: First, the bacteria were annotated according to the results of each taxon. Detailed information about the bacteria and the abundance distribution of the bacteria can be obtained. According to the abovementioned bacteria, the richness and uniform information of different bacteria in the intestinal tract of patients with TBI could be obtained by further detailed analysis of bacteria abundance and OTUs between the sleep disorder group and the normal sleep group in patients after TBI. Additionally, common and proprietary operable unit classification information (OTUs) of patients with sleep disorders and normal sleep is also available. Secondly, by comparing the fecal OTUs of patients with TBI with sleep disorders and normal sleep after TBI and using the form of a phylogenetic tree to analyze and compare, we determined whether there are significant differences in intestinal colony structure between different patients and different groups and to understand the specific differences. Furthermore, the experimental results were further analyzed and compared by principal component regression analysis (PCA). The experimental results are displayed by a dimensionality reduction map and a sample cluster tree.

After the analysis, to further understand the differences and specific structural differences of intestinal microflora between the sleep disorder group and the normal sleep group in patients after TBI, the statistical analysis method of the T-test was used to test the difference in fecal colony composition and specific colony results between the sleep disorder group and the normal sleep group. In addition, we can also understand the function of intestinal microflora between the two groups by analyzing the evolutionary relationship between the intestinal flora OTUs and the known bacteria in the literature and by further analyzing the functional differences.

## Results

### Operational taxonomic unit species composition analysis

**FIGURE 1:**
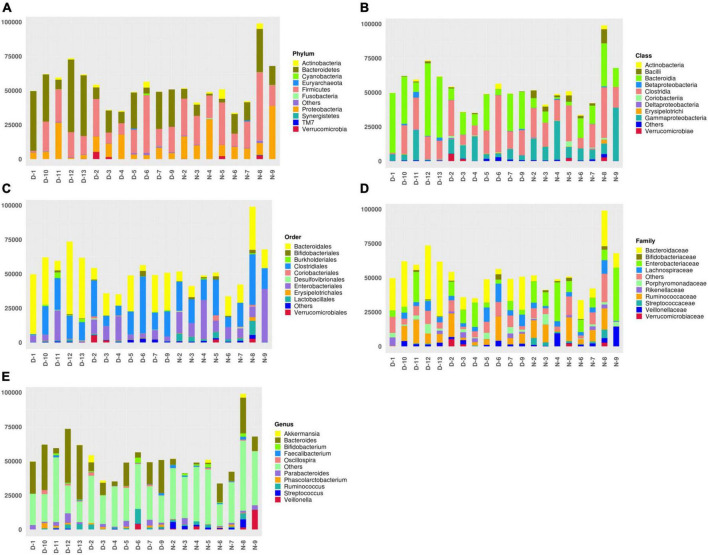
**(A)** The top 10 bacteria at the phylum level: Actinobacteria, Bacteroidetes, Cyanobacteria, Euryarchaeota, Firmicutes, Fusobacteria, Others, Proteobacteria, Synergistetes, TM7, and Verrucomicrobia. **(B)** The top 10 bacteria at the class level: Actinobacteria, Bacilli, Bacteroidia, Betaproteobacteria, Clostridia, Coriobacteriia, Deltaproteobacteria, Erysipelotrichi, Gammaproteobacteria, Others, and Verrucomicrobiae. **(C)** The top 10 bacteria at order level: Bacteroidales, Bifidobacteriales, Burkholderiales, Clostridiales, Coriobacteriales, Desulfovibrionales, Enterobacteriales, Erysipelotrichales, Lactobacillales, Others, and Verrucomicrobiales. **(D)** The top 10 bacteria at the family level: Bacteroidaceae, Bifidobacteriaceae, Enterobacteriaceae, Lachnospiraceae, Others, Porphyromonadaceae, Rikenellaceae, Ruminococcaceae, Streptococcaceae, Veillonellaceae, and Verrucomicrobiaceae. **(E)** The top 10 bacteria at genus level: Akkermansia, Bacteroides, Bifidobacterium, Faecalibacterium, Oscillospira, Others, Parabacteroides, Phascolarctobacterium, Ruminococcus, Streptococcus, and Veillonella.

According to the annotation results of different microflora, the top 10 bacteria in the fecal samples of patients with a sleep disorder or normal sleep after craniocerebral injury were selected in different classification levels (Phylum, Class, Order, Family, and Genus) to form the relative abundance map of varying flora and to further understand in detail the difference between each flora in different classification levels, as well as the bacteria with high relative content in different flora and their proportion. The top 10 bacteria at the phylum level are as follows: Actinobacteria, Bacteroidetes, Cyanobacteria, Euryarchaeota, Firmicutes, Fusobacteria, Others, Proteobacteria, Synergistetes, TM7, and Verrucomicrobia. The top 10 bacteria at the class level are Actinobacteria, Bacilli, Bacteroidia, Betaproteobacteria, Clostridia, Coriobacteriia, Deltaproteobacteria, Erysipelotrichi, Gammaproteobacteria, others, and Verrucomicrobiae. The top 10 bacteria at order level are Bacteroidales, Bifidobacteriales, Burkholderiales, Clostridiales, Coriobacteriales, Desulfovibrionales, Enterobacteriales, Erysipelotrichales, Lactobacillales, others, and Verrucomicrobiales. The top 10 bacteria at the family level are Bacteroidaceae, Bifidobacteriaceae, Enterobacteriaceae, Lachnospiraceae, others, Porphyromonadaceae, Rikenellaceae, Ruminococcaceae, Streptococcaceae, Veillonellaceae, and Verrucomicrobiaceae. The top 10 bacteria at the genus level are Akkermansia, Bacteroides, Bifidobacterium, Faecalibacterium, Oscillospira, others, Parabacteroides, Phascolarctobacterium, Ruminococcus, Streptococcus, and Veillonella, as shown in Figure 1.

### Operational taxonomic unit clustering and heatmap

**FIGURE 2:**
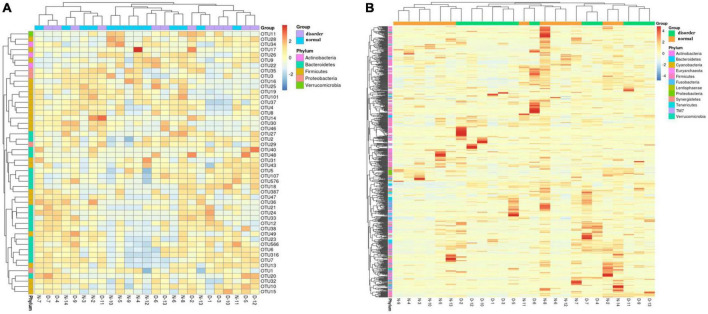
**(A)** Top 50 abundance OTUs cluster diagram. **(B)** All OTUs clustering diagrams.

According to the specific abundance information of different microflora in the sleep disorder group and the normal sleep group after TBI, the top 50 microflora information was further clustered and analyzed from the point of view of the number of samples of different flora information to make a thermograph. At the same time, combined with the phylum information annotated by different flora, the gate level distribution information of the normal sleep group and sleep disorder group after craniocerebral injury was analyzed by a cluster thermomap. The difference in intestinal flora between the two groups was observed, as shown in Figure 2 (the A, B, X, and Y axes are samples and OTU, respectively, and the color shade represents the OTU abundance. Cluster analysis of OTU and samples respectively; see the picture’s left and upper side of the picture. The color gradient can be customized to have two or more color gradients. The left and upper clustering trees are the clustering results of samples and OTUs, respectively. By analyzing the heat map of Figure 2, it was found that the differences in intestinal flora between the sleep disorder group and the normal sleep group after craniocerebral injury were as follows: Actinobacteria, Bacteroidetes, Firmicutes, and Verrucomicrobia, as shown in Figure 2.

### Inter-sample principal component analysis

**FIGURE 3:**
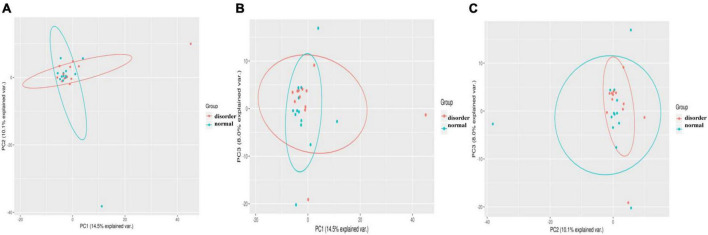
Samples from different environments may show dispersion and aggregation distribution, and the three components with the highest degree of explanation for sample differences in PCA results can be used to verify hypothetical factors. PC1 **(A)**, PC2 **(B)**, and PC3 **(C)** represent the suspected influencing factors of microbial composition deviation between sleep disorder group and normal sleep group, respectively, indicating that there are significant differences in microflora between sleep disorder group and normal sleep group after TBI.

This study should use the method of principal component analysis (PCA, principal component analysis) to find the difference in intestinal flora between the sleep disorder group and the normal sleep group after craniocerebral injury, remove the interference factors, simplify the initial complex data, and find the difference in intestinal flora between the sleep disorder group and the normal sleep group. The method is simple to operate and has no obvious limitations in terms of special parameters. By analyzing the differences and specific differences of intestinal microflora between the sleep disorder group and the normal sleep group, we described the difference in specific data of multiple groups of different microflora as a two-dimensional coordinate map by using the method of variance decomposition, which further reflects the characteristics of meaningful flora. For example, the smaller the difference in intestinal microflora between the sleep disorder group and the normal sleep group, the closer the curve distance of the microflora in the PCA diagram. Figure 3 shows a significant difference in microflora between the sleep disorder group and the normal sleep group after craniocerebral injury.

### Linear discriminant analysis effect size analysis

In this study, linear judgment analysis and influence factor (LEfSe, linear discriminant analysis Effect Size) were used to analyze the characteristics of different intestinal flora between the sleep disorder group and the normal sleep group after TBI, and the different degree of microflora between the two groups was described in detail.

In this study, LEfSe was mainly used to interpret further the differences in intestinal microflora between the sleep disorder group and the normal sleep group after TBI. LEfSe analysis has three main steps: the first step involved detecting the differential abundance of intestinal microflora between the sleep disorder group and the normal sleep group by a non-parametric Kruskal–Wallis (KW) sum-rank test. In the second step, the Wilcoxon rank-sum test was used to test whether the difference in differential bacteria in the previous step was consistent between the sleep disorder group and the normal sleep group. Finally, LDA linear discriminant analysis was used to estimate the effect of these differential bacteria on sleep disorders after TBI.

**FIGURE 4:**
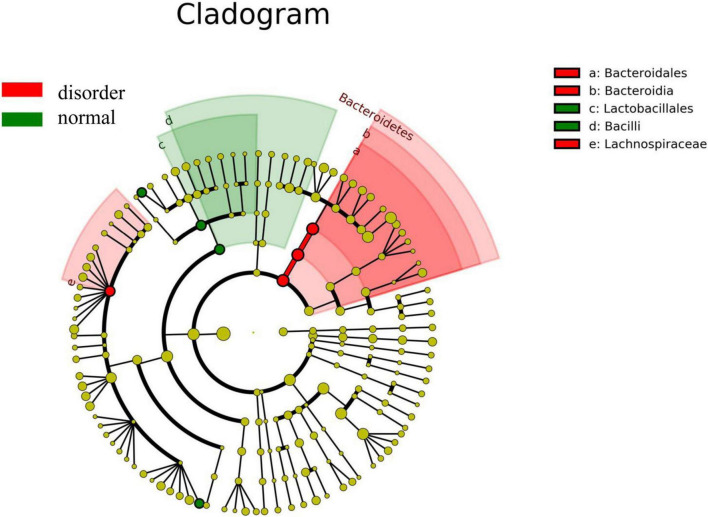
Group grouping comparison and analysis of the evolutionary cladistic map. The small circle radiating from inside to outside represents the species classification level of phylum, class, order, family, and genus, respectively, and the diameter of the small circle represents relative abundance. The different color nodes in the evolutionary tree are the microbial groups that play an essential role in this color group. The species name corresponding to the biomarker not shown in the figure is shown on the right, and the letter number corresponds to that in the figure. The intestinal flora of patients with normal sleep after TBI was mainly Bacilli and Lactobacillales, while that of patients with sleep disorders after TBI was mainly Lachnospiraceae and Bacteroidales.

**FIGURE 5:**
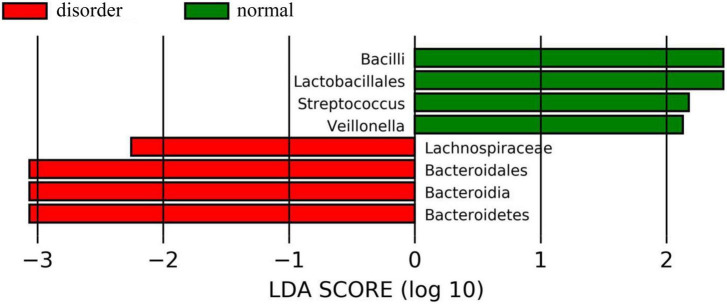
The histogram of group value distribution by grouping comparison and analysis. The ordinate is the biomarker with a statistical difference, and the abscissa is the LDA score. The higher the LDA score, the more significant the difference between groups. The color of the bar chart represents the respective groups. There were significant differences in intestinal flora between the normal sleep group and the sleep disorder group after TBI. Bacilli, Lactobacillales, Streptococcus, and Veillonella were dominant in the normal sleep group, and Lachnospiraceae, Bacteroidales, Bacteroidia, and Bacteroidetes were dominant in the sleep disorder group.

**FIGURE 6:**
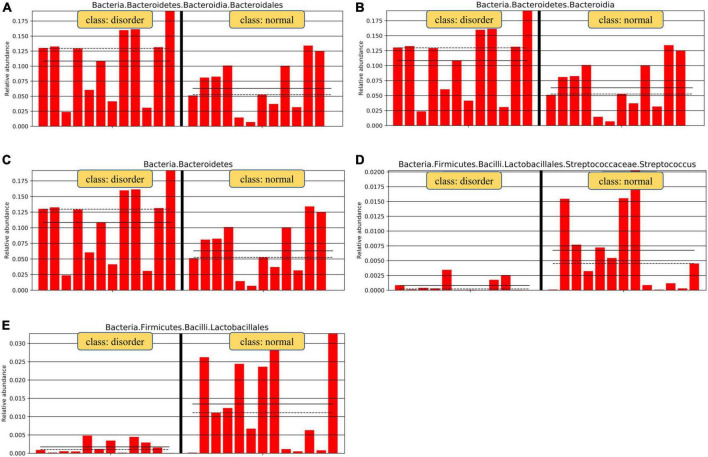
Relative abundance map of species with significant differences by group grouping comparison. There are significant differences in intestinal microflora between patients with sleep disorders after TBI and those with normal sleep after TBI. The main manifestations of intestinal flora are Firmicutes, Bacilli, Lactobacillales, Streptococcaceae, and Bacteroidetes. Analysis of microflora differences between groups.

Analysis software: LEfSe^1^ performs linear discriminant analysis (LDA) on samples according to different grouping conditions according to taxonomic composition to find out the species that have significant differences in sample division. Group grouping comparison and analysis of evolutionary cladistic maps showed the intestinal flora of patients with normal sleep after TBI was mainly Bacilli and Lactobacillales, while that of patients with sleep disorders after TBI was mainly Lachnospiraceae and Bacteroidales. The histogram of group value distribution by grouping comparison and analysis showed significant differences in intestinal flora between the normal sleep group and the sleep disorder group after TBI. Bacilli, Lactobacillales, Streptococcus, and Veillonella were dominant in the normal sleep group, and Lachnospiraceae, Bacteroidales, Bacteroidia, and Bacteroidetes were dominant in the sleep disorder group. A relative abundance map of species with significant differences by group grouping comparison showed significant differences in intestinal microflora between patients with sleep disorders after TBI and those with normal sleep after TBI. The main manifestations of intestinal flora are Firmicutes, Bacilli, Lactobacillales, Streptococcaceae, and Bacteroidetes, as shown in Figures 4–6.

## Discussion

Some scholars have recently found that the intestinal microflora is regulated by the hypothalamic-pituitary-adrenal axis (HPA). As a key regulator of the depression response system, intestinal microflora plays a key role in the progression of depression-related disorders, including posttraumatic stress disorder (PTSD) (Hemmings et al., 2017). The brain regulates intestinal function and counteracts the brain, and the brain-gut axis is the bridge between the two. The brain-gut axis is a two-way regulatory axis of the brain and gastrointestinal tract interaction. It includes the central nervous system (CNS), autonomic nervous system (ANS), intestinal nervous system (ENS), HPA axis, and other structures. The gastrointestinal tract is the only organ controlled by the CNS, ANS, and ENS, which has both sensory and motor functions. The relationship between posttraumatic sleep disorder and posttraumatic stress disorder (PTSD) is pervasive. At the same time, some studies have found that the disorder of the HPA axis has a certain effect on the pathophysiology of PTSD (Leichsenring et al., 2020). Combined with a previous study (Zhanfeng et al., 2019), sleep disorders in patients with craniocerebral injury were related to the disorder of central circadian biorhythm. We found that the expression of Per2, Clock, and Bmal1 in oral mucosal cells and peripheral blood monocytes in patients with sleep disorders after craniocerebral injury was significantly lower than that in patients with normal sleep after craniocerebral injury (Zhanfeng et al., 2019). The central biorhythm regulation center is located in the hypothalamic suprachiasmatic nucleus (SCN nucleus). Therefore, this part explores whether a sleep disorder complicates intestinal flora disorders in patients with craniocerebral injury and finds the meaningful intestinal flora of patients with sleep disorders after craniocerebral injury. This study aims to explore the relationship between central biorhythm and intestinal flora in the later stages and to provide a preliminary theoretical basis for whether it can change intestinal flora and sleep disorder symptoms in patients with craniocerebral injury.

Intestinal microflora usually refers to microbes with genetic information in the gastrointestinal tract. At present, there are more than 1,000 kinds of microbes in the intestinal tract of adults. The largest number of species belong to Firmicutes and Bacteroidetes. The following five species belong to Proteobacteria, Fusobacteria, Verrucomicrobia, and Cyanobacteria (Finegold et al., 2010; Adams et al., 2011; Iglesias-Vázquez et al., 2020). When a person is healthy, the composition of these bacteria is in a state of dynamic equilibrium. The microbalance of intestinal flora is crucial for maintaining patients’ health because the imbalance of intestinal flora significantly increases the host’s susceptibility to disease (Falk et al., 1998).

The main purpose of this study is to detect the structure of fecal microflora in patients with craniocerebral injury complicated by sleep disorders and normal sleep after craniocerebral injury by 16S-rDNA sequencing. There were significant differences in intestinal flora between the normal sleep group and the sleep disorder group after craniocerebral injury. Bacilli, Lactobacillus, Streptococcus faecalis, and Veronella were dominant in the normal sleep group, while Lachnospiraceae, Bacteroidales, Bacteroidia, and Bacteroidetes were dominant in the sleep disorder group. We can find a significant difference in intestinal flora between the sleep disorder and the normal sleep groups after craniocerebral injury. Under the premise of the same degree of craniocerebral injury, the difference in intestinal flora is related to sleep disorder after craniocerebral injury. Therefore, intestinal flora imbalance occurs in patients with sleep disorders after craniocerebral trauma. Intestinal flora regulation can be used as a therapeutic target to regulate sleep in patients after craniocerebral injury in follow-up studies.

It was found that there were some changes in the number and species of intestinal bacteria in the sleep disorder group after craniocerebral injury compared with the normal sleep group after craniocerebral injury. The numbers of Lactobacillus, Streptococcus, Oribacterium, Rothia, Actinomyces, Streptophyta, TM7-3, and Serratia decreased significantly, while the numbers of Odoribacter, Lachnospiraceae, and Bilophila increased significantly. It also showed that the intestinal flora decreased, and the number of microflora increased considerably in patients with sleep disorders after craniocerebral injury. In recent years, studies have found that intestinal microflora shows an obvious biological rhythm in flora structure and function. For example, Clostridiales (Clostridium), Lactobacillales (Lactobacillus), and Bacteroidales (Bacteroides), which account for approximately 60% of the intestinal flora, have obvious rhythms, causing obvious special characteristics of time classification (Krueger and Opp, 2016). As we selected patients with sleep disorders after craniocerebral injury for fecal flora analysis, we found that the number of Lactobacillus with biorhythmic expression decreased significantly in the sleep disorder group. In the first part, we found the biorhythm expression disorder in the sleep disorder group, indicating a correlation between Lactobacillus and central biorhythm expression; whether there is consistency between the two needs further study. Liang (Brooks et al., 2021) also found that two important bacteria in the intestinal tract of mammals are Bacteroidetes and Firmicutes, which show obvious rhythmic changes from day to night in terms of the periodic changes of flora. These changes are not only due to the adjustment of diet and diet but also related to the biological clock and the sex of the host. In the study of Benedict (Benedict et al., 2016), it is discussed that Lachnospiraceae also belong to Firmicutes, changing Lachnospiraceae in this study, which is consistent with the results of previous studies. Therefore, in this group of patients, it has been found that the expression abundance of Bacteroides and Lachnospiraceae is higher in the sleep disorder group, which may play a certain role in regulating sleep in patients after craniocerebral injury. Some studies have found that human biological clock disorders, sleep disorders, and night shifts can cause changes in central biological clock genes and intestinal microbial colony structure (Kesika et al., 2021). We infer that sleep disorders in patients after craniocerebral injury affect the structure of intestinal flora and disturb the intestinal flora, both of which are regulated by the brain-gut axis. Intestinal dysfunction occurs in patients with craniocerebral injury and vice versa.

Until now, a large number of studies have shown that there is a microbial-intestinal-brain axis in the human body (Montiel-Castro et al., 2013; Cryan et al., 2019). The enterocerebral axis plays a crucial role in the pathogenesis and development of many diseases, such as depression, schizophrenia, and autism (Molina-Torres et al., 2019). Intestinal microorganisms can produce bidirectional information through this axis with host immune cells, neuroendocrine pathways, and vagus pathways to affect brain function (Wang and Wang, 2016). In a small human study focusing on non-combat posttraumatic stress disorder, the relative abundance of actinomycetes, chronic sporomycetes, and Verrucomicrobia (verrucous microflora) in posttraumatic stress disorder was lower than that in the control group exposed to trauma (Forehand et al., 2019). We found that the abundance of Lactobacillus, Streptococcus, Oribacterium, Rothia, Actinomyces, Streptophyta, TM7-3, and Serratia decreased significantly in patients with sleep disorders after craniocerebral trauma. Compared with patients with normal sleep after craniocerebral trauma, sleep disorder could be considered an independent factor for decreasing intestinal flora abundance. At the same time, this study found that the abundance of intestinal flora decreased in patients with sleep disorders after craniocerebral trauma, but the abundance of Odoribacter, Lachnospiraceae, and Bilophila increased relatively. It was found that Lachnospiraceae belongs to the genus containing short-chain fatty acids, which can strengthen the intestinal barrier and have a better curative effect on patients with intestinal diseases (Bajaj et al., 2015). Although these findings do not explain how they affect posttraumatic sleep disorders, they emphasize the role of intestinal microflora in patients with posttraumatic sleep disorders. We further studied the relationship between sleep disorder and central biological clock gene expression and intestinal flora in patients after craniocerebral injury to improve patients’ clinical symptoms by regulating intestinal flora to affect brain function.

The study of mice found that sleep disorders in mice can affect the structure and diversity of intestinal flora (Zhang et al., 2017). These findings suggest that biological clock genes may affect intestinal microflora. Similarly, intestinal microorganisms also show biorhythm oscillations in terms of colony composition and function. Therefore, intestinal mucosal epithelial cells came into direct contact with a variety of different colonies, and their metabolites ran through the whole body all the time. On the contrary, the biorhythm changes of intestinal flora led to changes in the host central biorhythm. We found a significant difference in intestinal flora disorder between the sleep disorder group and the normal sleep group after craniocerebral injury. Combined with the first part of the study, it was found that the expression of biorhythm was disordered in the sleep disorder group. Therefore, we believe that the intestinal flora and biorhythm of patients with sleep disorders are disrupted, but additional animal tests are required to determine whether they influence each other or not. Therefore, this topic explains that there may be a relationship between intestinal microbial colonies and host central biorhythm in patients after craniocerebral injury. How to explore this relationship in depth is the focus of our further research to solve the problems caused by long-term sleep disorders.

There may be an interaction between intestinal microflora and sleep disorders, and inflammation and endocrine hormones play an essential role in the whole process. First, sleep disorders and central circadian disorders in patients after craniocerebral injury affect the metabolism of inherent intestinal microflora and cause changes in the structure of intestinal microflora, especially by reducing the overall number of Lactobacillus. It can increase the overall number of Bacteroides, Enterococci, and Spirulina, which cause intestinal flora dysfunction. Second, intestinal microecology regulates the interaction of intestinal mucosal cells by reducing intestinal mucosal permeability and protecting the intestinal barrier. Once the intestinal barrier is broken, the intestinal flora and its metabolites enter the intestinal mucosa, activate the inflammatory response system, and further stimulate the vagus nerve and spinal cord afferent nerve. The possible mechanism is that the imbalance of intestinal flora causes inflammation, which further affects the central nervous system and causes or exasperates sleep disorders in patients after craniocerebral injury.

Furthermore, the intestinal microflora makes a connection with the expression of biological clock genes. Therefore, we need to study further how the biorhythmic activity of intestinal flora is involved in the regulation of central biorhythm or intestinal biorhythm. It is also necessary to clarify how biorhythm concussion and functional activity are regulated between intestinal flora and sleep disorders after craniocerebral injury and whether the microbial community can change the host biorhythm.

## Conclusion

The main purpose of this study is to detect the structure of fecal microflora in patients after craniocerebral injury, which is complicated by sleep disorders and normal sleep, by 16S-rDNA sequencing. There were significant differences in intestinal flora between the normal sleep group and the sleep disorder group after craniocerebral injury. It is suggested that the sleep disorder in patients after craniocerebral injury may be closely related to the disturbance of intestinal flora related to the brain-gut axis, and it can be inferred that the regulation of intestinal flora may be a new therapeutic target for sleep disorders after craniocerebral injury.

## Data availability statement

The datasets presented in this study can be found in online repositories. The names of the repository/repositories and accession number(s) can be found below: BioProject ID: PRJNA839530.

## Ethics statement

The studies involving human participants were reviewed and approved by Ethics Committee of General Hospital of Ningxia Medical University. The patients/participants provided their written informed consent to participate in this study.

## Author contributions

NZ and XH were responsible for the project design and experimental step guidance. NZ mainly wrote the manuscript. NZ, WL, and KJ were responsible for specific experimental content and data analysis and processing. BJ and CY were responsible for collecting patient information for the group. All authors contributed to the article and approved the submitted version.

## Footnotes

1
http://huttenhower.sph.harvard.edu/galaxy/root?tool_id=lefse_upload
