# Supplementary material for: Regulation of sleep disorders in patients with traumatic brain injury by intestinal flora based on the background of brain-gut axis
Source: Front Neurosci. 2022 Oct 11;16:934822. doi: 10.3389/fnins.2022.934822 (PMC9594989; doi:10.3389/fnins.2022.934822)
Supplement: Supplementary file 1 [file Table_1.DOCX]

**Supplementary Table 1. Sleep disorder and Normal sleep Patients Characteristics**

| **Patients Characteristics** | **Sleep disorder group** | **Normal sleep disorder** | *P* **Value** |
| --- | --- | --- | --- |
| **n** | **14** | **14** |  |
| **Sex (Male/Female)** | **11/3** | **11/3** |  |
| **Age (years)** | **41.86±14.28(18-69)** | **41.64±12.31(20-57)** | ***P>0.05(P=0.8421)*** |
| **Weight(Kg)** | **66.93±11.35(52-84)** | **67.21±11.80(49-86)** | ***P>0.05(P=0.7315)*** |
| **systemic diseases(such as heart,liver or kidney)** | **0** | **0** |  |
| **GCS(score)** | **14.21±0.80** | **14±0.78** | ***P>0.05(P=0.5171)*** |
| **Marshal CT classification** |  |  | ***P>0.05(P=1)*** |
| **The Length of Hospital Stay (Days)** |  |  | ***P>0.05(P=0.7659)*** |
| **Sleep disorder time(days)** | **42.21±9.06** | **2.36±1.39** | ***P< 0.0001*** |
| **PQSI (total score)** | **18.14±1.46** | **2.43±1.34** | ***P< 0.0001*** |
| **Sleep efficiency(EEG)** | **9.53±3.57** | **7.35±0.79** | ***P>0.05(P=0.1562)*** |
