# Supplementary material for: Regulation of sleep disorders in patients with traumatic brain injury by intestinal flora based on the background of brain-gut axis
Source: Front Neurosci. 2022 Oct 11;16:934822. doi: 10.3389/fnins.2022.934822 (PMC9594989; doi:10.3389/fnins.2022.934822)
Supplement: Supplementary file 2 [file Table_2.docx]

**Supplementary Table 2. PSQI score and sleep time of each patient**

| **Number of Patients** | **Sleep disorder** | | **Normal sleep** | |
| --- | --- | --- | --- | --- |
|  | **PSQI(Score)** | **Sleep time**  **(hour)** | **PSQI(Score)** | **Sleep time**  **(hour)** |
| **1** | 17 | 5.67 | 5 | 8 |
| **2** | 19 | 4.5 | 3 | 7 |
| **3** | 21 | 12 | 2 | 8 |
| **4** | 18 | 13.2 | 1 | 6.5 |
| **5** | 18 | 6 | 2 | 7 |
| **6** | 17 | 3.5 | 0 | 9 |
| **7** | 16 | 7.5 | 1 | 7.5 |
| **8** | 19 | 13 | 2 | 7.5 |
| **9** | 20 | 5.5 | 4 | 8 |
| **10** | 17 | 14 | 2 | 6 |
| **11** | 16 | 15 | 3 | 7 |
| **12** | 18 | 4.6 | 4 | 7 |
| **13** | 19 | 12 | 2 | 6.5 |
| **14** | 19 | 12.5 | 3 | 8 |

**evaluation degree of PSQI: Score 0-5 Good sleep quality**

**6-10 Fine sleep quality**

**11-15 Common sleep quality**

**16-21 Poor sleep quality**
